# Associations Between Perinatal Dioxin Exposure and Circadian Clock Gene mRNA Expression in Children in Dioxin-Contaminated Areas of Vietnam

**DOI:** 10.3390/toxics13030191

**Published:** 2025-03-07

**Authors:** Thao Ngoc Pham, Hoa Thi Vu, Takafumi Tasaki, Tai Pham-The, Nghi Ngoc Tran, Muneko Nishijo, Tien Viet Tran, Hai Anh Tran, Tomoya Takiguchi, Yoshikazu Nishino

**Affiliations:** 1 Department of Functional Diagnosis, Military Hospital 103, Vietnam Military Medical University, Hanoi 12108, Vietnam; phamngocthaovmmu@gmail.com; 2 Department of Military Hygiene, Vietnam Military Medical University, Hanoi 12108, Vietnam; vuhoa5593hvqy@gmail.com; 3 Department of Life Science, Medical Research Institute, Kanazawa Medical University, Ishikawa 920-0293, Japan; tasakit@kanazawa-med.ac.jp; 4 Biomedical and Pharmaceutical Research Centre, Vietnam Military Medical University, Hanoi 12108, Vietnam; taithuy@kanazawa-med.ac.jp; 5 Ministry of Health, Vietnamese Government, Hanoi 10060, Vietnam; tranngocnghibyt@gmail.com; 6 Epidemiology and Public Health, Kanazawa Medical University, Ishikawa 920-0293, Japan; ttakiguc@kanazawa-med.ac.jp (T.T.); ynishino@kanazawa-med.ac.jp (Y.N.); 7 Department of Infectious and Tropical Diseases, Military Hospital 103, Vietnam Military Medical University, Hanoi 12108, Vietnam; tientv@vmmu.edu.vn; 8 Department of Physiology, Vietnam Military Medical University, Hanoi 12108, Vietnam; anhhtr@yahoo.com

**Keywords:** dioxin, clock gene, circadian rhythm, behavior problems, Vietnam

## Abstract

We investigated the impact of perinatal dioxin exposure (indicated by dioxin levels in maternal breast milk) on clock gene mRNA expression in buccal cells of 9-year-old children from the Da Nang birth cohort in Vietnam using reverse transcription polymerase chain reaction. Of the 56 boys and 34 girls (67% detection rate) in whom *PER1* was detected, *BMAL1* was detected in only 16 boys and 15 girls. Dioxin levels were significantly higher in girls with *BMAL1* detection than in girls without detection. In girls, higher relative *BMAL1* expression levels were associated with greater levels of 2,3,7,8-tetrachlorodibenzo-p-dioxin and toxic equivalents of polychlorinated dibenzodioxins and polychlorinated dibenzofurans. Moreover, *BMAL1* expression levels were correlated with shorter night sleep duration on weekdays, greater sleep duration on holidays, and higher hyperactivity scores. After adjusting for maternal parity, relative *PER1* expression levels were higher in boys with higher toxic equivalents of polychlorinated dibenzofuran than those in girls. Although higher *PER1* expression levels were correlated with greater verbal aggression and hostility scores in girls, no such associations were found in boys. These findings suggest the possible existence of sex-specific effects of perinatal dioxin exposure on circadian rhythms regulated by clock genes, particularly *BMAL1*, leading to sleep and behavioral problems in later life.

## 1. Introduction

Circadian rhythms are 24-h cycles that coordinate biological functions and prepare the body for recurring diurnal activities, such as sleeping, eating, and physical activity [1,2]. Increasing evidence indicates that chemical contamination (such as dioxin) of the environment can disrupt circadian rhythms, which are associated with adverse health outcomes. In a previous study, Miller et al. (1999) reported that 2,3,7,8-tetrachlorodibenzo-p-dioxin (TCDD) exposure disrupts circadian-controlled rest/activity cycles [3]. Moreover, Fader et al. (2019) reported that TCDD exposure induces alterations in the rhythmicity of several core clock regulators [4]. Similarly, TCDD exposure reportedly changes the expression of core circadian genes in the hypothalamus and decreases phase shifts in response to light [5]. However, these studies used animal models [3,4,5], and epidemiological studies investigating the impact of dioxin exposure on human circadian rhythms remain limited.

In dioxin-contaminated areas in Da Nang, Vietnam, we recruited 241 mother–child pairs in 2008–2009 and followed them up to investigate the adverse effects of perinatal dioxin exposure—indicated by dioxin levels in maternal breast milk—on different aspects of neurodevelopment at several ages. An association between higher TCDD exposure and increased autistic traits was identified in both boys and girls at 3 years of age. The boys also exhibited poor language and motor development associated with high levels of toxic equivalent (TEQ) polychlorinated dibenzodioxins and polychlorinated dibenzofurans (PCDD/Fs) [6]. At 5 years of age, perinatal dioxin exposure to TCDD was associated with increased hyperactivity scores in boys. When the children were 8 years old, significantly increased hyperactivity scores on the attention deficit hyperactivity disorder (ADHD) rating scale (RS) were associated with higher TCDD and TEQ-PCDD/Fs levels in girls but not in boys [6]. At the same age, covert aggression scores on the Children’s Scale of Hostility and Aggression: Reactive/Proactive (C-SHARP) were significantly higher with higher TCDD levels, particularly in girls [6]. These findings indicate that increased behavioral problems, such as hyperactivity and aggression, which are often observed in children with ADHD, may be associated with perinatal dioxin exposure, particularly in girls.

Abnormal circadian rhythms leading to altered sleep–wake cycles are often observed in children with neurodevelopmental disorders such as autism spectrum disorder or ADHD [7,8]. In children with ADHD, previous studies [8,9] have indicated that alterations in the circadian system may be a core pathology of the disease. These findings motivated us to investigate the effects of perinatal dioxin exposure on circadian rhythm and sleep duration in children with ADHD traits from the Da Nang cohort in Vietnam.

To investigate alterations in circadian rhythms, measurements of the mRNA expression of core clock genes (such as *BMAL1* and *CLOCK*) from saliva or buccal mucosa samples are commonly used because of the circadian expression of clock gene mRNA and proteins that regulate salivary excretion [10]. In addition, mRNA analyses using saliva or buccal mucosa are non-invasive and relatively easy to perform, especially if an RNA collection kit with an RNA stabilizer is used. This makes it relatively simple to conduct epidemiological studies on children at local survey sites.

We therefore collected buccal mucosa from 9-year-old children in the Da Nang birth cohort. We then investigated the associations between perinatal dioxin exposure and mRNA expression levels of the clock genes *BMAL1*, *PER1*, *CRY1*, and *CRY2* in the buccal mucosa. Next, we analyzed the associations between clock gene mRNA expression levels and symptoms of ADHD, including alterations in sleep duration, to explore whether the disruption of circadian rhythms due to dioxin exposure during the perinatal period results in behavioral problems later in life.

## 2. Materials and Methods

### 2.1. Participants and Study Area

In 2008 and 2009, we recruited 241 mother–child pairs living in the Thanh Khe and Son Tra districts of Da Nang city, Vietnam. This area is within 10 km of the former United States Da Nang airbase, and the mothers gave birth in the obstetric departments of two district hospitals. To evaluate exposure markers in infants, dioxins in maternal breast milk were measured, and high levels were reported in our previous study [11]. We then followed up with these children to examine their neurodevelopment at various periods from 4 months to 8 years [6,12,13].

In 2017–2018, 135 children (79 boys and 56 girls) participated in the current 9-year-old-follow-up survey to measure clock gene expression in buccal mucosa samples and interview about sleep (56% of follow-up rate). No significant differences in dioxin exposure levels, birth weight, gestational weeks at birth, or maternal factors were found between the 135 children who participated and the 106 children lost to follow-up.

Because RNA collection did not always yield sufficient samples for clock gene analysis and there were some failures for buccal sample collection by an inexperienced examiner, the number of children in the final data analysis was 90 (56 boys and 34 girls) in the present study. Before analyzing the data, we confirmed no significant differences in dioxin exposure levels and maternal and infant factors at birth between the 90 children who participated in the current analysis and the 45 children excluded from the analysis.

Information about the mothers and their families—such as maternal age, education, parity categories, alcohol consumption during pregnancy, smoking habits of the mother and family members, family income, and infant weight and gestational weeks at birth—were collected in our previous studies at birth and 4 months of infant age. In the present survey, at 9 years of age, the body size (including height, weight, and abdominal circumference) of the children was measured; their mean values are shown together with information from birth in Table 1.

Written informed consent was obtained from all mothers or caregivers according to a process that was reviewed and approved by the Health Department of Danang City and the Vietnam Military Medical University. The Institutional Ethics Board for Epidemiologic Studies at Kanazawa Medical University approved the study design (I-183, July 2017).

### 2.2. Dioxin Measurement

At 1 month after birth, a midwife or medical staff member from community health stations visited the mother’s house and collected maternal breast milk samples. Approximately 10 mL of breast milk was used to measure seventeen 2,3,7,8-substituted congeners of PCDDs and PCDFs using a gas chromatograph (HP-6980; Hewlett-Packard, Palo Alto, CA, USA) equipped with a high-resolution mass spectrometer (MStation-JMS700, JEOL, Tokyo, Japan). The TEQs of PCDDs, PCDFs, and PCDD/Fs were calculated with reference to the World Health Organization 2005 TEQ factors [14]. The established method of analysis has been described in detail in a previous study [11]. We used TCDD concentrations and TEQs of PCDDs, PCDFs, and PCDD/Fs as dioxin exposure markers in the present study.

### 2.3. Quantification of Clock Genes Using Reverse Transcription Polymerase Chain Reaction

On the examination day, the children were asked to clean their mouths using a bottle of water and were told not to eat or drink for at least 30 min prior to buccal cell collection. A cotton-tipped swab (Puritan Medical Products, Guilford, ME, USA) was used to collect buccal cells along the inside of the cheek, which were then placed into an RNA collection tube (Mawi DNA Technologies LLC., Hayward, CA, USA). An RNeasy Mini Kit (Qiagen, Hilden, Germany) was used following the manufacturer’s instructions, with modifications to improve RNA quality. To extract total RNA, 500 μL of 95% ethanol was added to 250 μL of the sample, and the solution was incubated at −20 °C for 30 min. The solution was then centrifuged at 14,000× *g* for 3 min, and the supernatant was carefully removed to avoid disturbing the pellet. Next, 5 μL of carrier RNA was added before the cells were lysed using 350 μL of buffer RLT containing 1% 2-mercaptoethanol. Each sample was subsequently homogenized using a QIAshredder (Qiagen), and 350 μL of 70% ethanol was added and mixed thoroughly. The sample was then transferred to an RNeasy column and centrifuged at 8000× *g* for 30 s. After discarding the flow-through, 80 μL of DNase was added to the spin column surface of the RNeasy column and incubated at room temperature for 15 min. After cleaning 3 times, the RNeasy column was then moved to a new 2-mL tube and centrifuged at 10,000× *g* for 5 min with the lid open, to dry the RNeasy membrane. After the RNeasy column was moved to a new 1.5-mL tube, 25 μL of RNase-free water was added to the center of the membrane and incubated at room temperature for 5 min. The lid was then closed, and the solution was centrifuged at 12,000× *g* for 1 min to collect the RNA samples.

cDNA synthesis was performed using the Rever Tra Ace TM qPCR RT Master Mix with gDNA remover kit (TOYOBO CO., LTD., Osaka, Japan). We placed 2 μL of the solution of 4 × DN Master mixed with gDNA remover (1 μL gDNA remover mixed with 50 μL 4 × DN Master) with a 4 × DN Master/gDNA remover ratio of 220 μL/4.4 μL and 6 μL of RNA samples into two tubes. The two tubes were then placed in a block heating machine at 37 °C for 5 min. Next, 2 μL of 5× RT Master Mix II was added to one tube, and 2 μL of 5× RT Master Mix II and no-RT control were added to another tube. The samples were incubated at 37 °C for 15 min, 50 °C for 5 min, and 98 °C for 5 min before adding 20 μL of RNAse-free water.

Each 20 µL reaction mixture contained 10 μL of TaqMan 2× PCR Master Mix solution, 7 μL of RNAse-free water, 1 μL of Taqman probes for clock genes, and 2 μL of cDNA sample, and was analyzed using a Quant Studio 12 K Flex Real-Time PCR System (Thermo Fisher Scientific, Waltham, MA, USA). Four major clock genes (*BMAL1*, *PER1*, *CRY1*, and *CRY2*) and one reference gene (*ACTB*) were analyzed. TaqMan probes for these genes were purchased from Thermo Fisher Scientific. The results with mean cycle threshold (Ct) values lower than the detection limit of Ct = 38 were used for data analysis. Using the comparative Ct method (∆∆Ct), relative gene expression levels were calculated from the mean Ct values for the target and reference genes and used for data analysis.

### 2.4. Sleep Duration and Behavioral Assessments

In the present survey, children were interviewed about how long they napped and slept at night on weekdays and holidays. The most common nap, sleep at night, and total sleep (nap + sleep at night) durations on weekdays and holidays were used for the analysis.

When the children were 8 years of age, we interviewed parents (or caregivers who knew the children well) to evaluate the children’s behavioral disorders across two subscales: an inattention score (inattention) and an impulsivity and hyperactivity score (hyperactivity), as well as a total scale score (ADHD) using the ADHD-RS. For analysis, the standardized scores for each scale were calculated with reference to the percentiles in the score sheets, with a range of 1–99 percentiles [15]. At the same time, we examined the children’s aggressive behavior by interviewing parents or caregivers using the C-SHARP scale, which has five subscales (verbal aggression, bullying, covert aggression, hostility, and physical aggression) [16]. The detailed methods and results of these scales have been reported in our previous publications [6,12].

### 2.5. Statistical Analysis

SPSS for Windows (version 21.0; IBM Corp., Armonk, NY, USA) was used for statistical analysis. The levels of TCDD and TEQs of PCDDs, PCDFs, and PCDD/Fs in breast milk, as well as the relative gene expression of *BMAL1*, *PER1*, *CRY1*, and *CRY2*, were log_10_ transformed to normalize the data distribution for statistical analysis. Independent sample *t*-tests were used to compare the levels of TCDD and TEQs of PCDDs, PCDFs, and PCDD/Fs between groups with and without *BMAL1* detection. Spearman’s correlation test was used to analyze correlations between relative gene expression levels and dioxin levels, sleep durations, and ADHD-RS and C-SHARP scores. In the analysis of cases in which *PER1* was detected, a linear regression model was used after adjusting for confounding factors such as maternal parity or education to analyze the associations between clock gene expression and dioxins or neurodevelopmental indices.

## 3. Results

### 3.1. Characteristics of the Subjects and Sleep Duration

The mean birth weights and gestational ages were 3304.5 g and 39.5 weeks for boys and 3113.2 g and 39.5 weeks for girls, indicating normal birth weights and full-term births in most cases. The maternal ages for boys and girls were 28.9 and 27.8 years, respectively. The rate of primiparous mothers was higher in girls than in boys (35.1% vs. 19.7%); however, no significant differences in age were observed between the mothers of boys and girls. The years of school education of the mothers—8.8 years for boys and 8.2 years for girls—did not differ significantly. The mean body mass index levels for boys and girls were 18.3 and 17.3, which are within the normal range.

The mean sleep duration at night, nap duration, and total sleep duration on weekdays and holidays are shown in Table 1. On weekdays, nap durations were significantly shorter in girls than in boys; however, there was no significant difference in sleep duration at night. By contrast, on holidays, sleep duration at night was significantly longer in girls than in boys, whereas there was no significant difference in nap duration between the two groups.

### 3.2. Relative BMAL1 Expression Levels and Perinatal Dioxin Exposure

*BMAL1* was only detected (Ct value < 38) in samples from 31 children (16 boys and 15 girls), although *PER1* was detected in samples from 90 children (56 boys and 34 girls).

To investigate the influence of perinatal dioxin exposure on relative *BMAL1* expression, we first compared the mean levels of perinatal dioxin exposure markers between the groups with and without *BMAL1* detection using the *t*-test (Table 2). In boys, no significant differences in dioxin levels were observed between the *BMAL1*-detected and *BMAL1*-non-detected groups. In girls, however, the TEQ-PCDD/Fs levels were significantly higher in the *BMAL1*-detected group than in the *BMAL1*-non-detected group, which was attributed to higher levels of TCDD and PCDF congeners (Table 2).

Using only the cases with *BMAL1* detection, we analyzed the correlations between the levels of TCDD, TEQs of PCDDs, PCDFs, and PCDD/Fs, and the relative expression levels of *BMAL1*, *PER1*, *CRY1*, and *CRY2*. The correlation coefficients (Spearman’s rho) are shown in Table 3. Although no significant correlations between dioxin levels and clock gene expression were identified in boys, *BMAL1* expression levels had significant positive correlations with TCDD and TEQ-PCDD/F levels in girls. Furthermore, *PER1* expression levels were significantly and inversely correlated with TEQ-PCDF levels in girls (Table 3).

### 3.3. Associations Between Relative BMAL1 Expression Levels and Neurodevelopmental Symptoms, as Indicated by Sleep Durations and ADHD-RS and C-CHARP Scores

Because clock genes regulate the sleep–wake cycle, correlations between relative clock gene expression levels and sleep duration were analyzed using Spearman’s correlation tests among children with detected *BMAL1*. The results for sleep duration on weekdays and holidays, according to sex, are shown in Table 4. In boys, there were no significant correlations between sleep indicators (night sleep duration, nap duration, or total sleep duration) and relative clock gene expression levels in boys. In contrast, in girls, *BMAL1* expression levels had significant inverse correlations with night sleep and total sleep duration on weekdays. *CRY1* expression levels also had a significant inverse correlation with night sleep duration on weekdays; however, no significant correlation was observed with total sleep duration, possibly because of the positive (but non-significant) correlation with the nap duration. *CRY2* expression levels showed a significant positive correlation with nap duration but not with night sleep duration on weekdays (Table 4). Furthermore, on holidays, *BMAL1* expression was correlated with night sleep duration, suggesting a longer sleep (late wake-up) on days without school in girls with higher *BMAL1* expression.

To investigate the association between clock gene expression and neurodevelopmental symptoms, we analyzed the correlations between relative clock gene levels and ADHD-RS and C-SHARP scores in children with *BMAL1* detection (Table 5). In boys, *CRY1* expression levels were significantly correlated with physical aggression scores in the C-SHARP, and *CRY2* levels were significantly correlated with inattention scores in the ADHD-RS. In girls, *BMAL1* expression levels were significantly correlated with hyperactivity scores on the ADHD-RS, and *PER1* levels had a significant inverse correlation with hyperactivity scores. No significant correlations between any clock gene expression levels and C-SHARP scores were identified in girls (Table 5).

### 3.4. Adjusted Associations Between Relative Expression Levels of Clock Genes Other than BMAL1 and Perinatal Dioxin Exposure Levels in Children with PER1 Detection

We were only able to analyze simple correlations between relative clock gene expression levels and perinatal dioxin exposure because of the relatively small number of cases with *BMAL1* detection. We therefore analyzed their associations in all children with *PER1* detection after adjusting for maternal parity (primipara/multipara) as a confounding factor. In Table 6, Spearman’s rho and standardized correlation coefficient beta (adjusted for maternal parity) between *PER1*, *CRY1*, and *CRY2* expression levels and the levels of TCDD and the three TEQs are shown according to sex. In boys, *PER1* expression levels showed a significant correlation with TEQ-PCDFs; this finding was significant even after adjusting for maternal parity. However, no significant correlations were identified between the other dioxin indices and *PER1*, *CRY1,* or *CRY2* expression levels. In girls, *PER1* expression levels had a significant inverse correlation with TEQ-PCDFs and TEQ-PCDD/Fs. However, after adjusting for maternal parity, the inverse correlation between *PER1* levels and TEQ-PCDFs remained significant, whereas the correlations between *PER1* levels and TEQ-PCDD/Fs were not significant. No significant correlations were observed between *CRY1* or *CRY2* levels and any dioxin indices in girls or boys (Table 6).

### 3.5. Adjusted Associations Between Relative Expression Levels of Clock Genes Other than BMAL1 and Neurodevelopmental Symptoms Indicated by Sleep Durations and ADHD and C-SHARP Scores in Children with PER1 Detection

Because there were no significant correlations between *CRY1* and *CRY2* levels and perinatal dioxin exposure (Table 6), we analyzed the associations between *PER1* expression levels and neurodevelopmental symptoms in children with *PER1* detection, after adjusting for the maternal education period. Table 7 shows the Spearman’s rho and standardized beta results of the ADHD-RS and C-SHARP scores only, because there were no significant associations between *PER1* levels and sleep duration on weekdays or holidays.

In girls only, *PER1* expression levels showed significant correlations with verbal aggression and hostility scores in the C-SHARP, even after adjusting for maternal education levels (Table 7). This finding suggests that increased *PER1* expression may increase verbal aggression and hostility in girls exposed to PCDF congeners at lower TEQ levels during the perinatal period.

## 4. Discussion

### 4.1. Impacts of Perinatal Dioxin Exposure on Clock Gene Expression Levels

In the present study, relative *BMAL1* mRNA expression was detected in the buccal cells of only 31 children (34% of those with *PER1* detection). However, our results indicate that, in girls, mean perinatal dioxin exposure levels (including TCDD) were 1.5 to 1.8 times higher in the *BMAL1*-detected group than in the *BMAL1*-non-detected group. Among the girls with *BMAL1* detection, levels of TCDD and TEQ-PCDD/Fs were positively correlated with *BMAL1* expression levels. In contrast, only TEQ-PCDF levels were correlated with relative *PER1* expression levels, suggesting that girls specifically exposed to high levels of PCDFs had lower *PER1* expression levels. These results indicate that TCDD and TEQ-PCDD/Fs originating from Agent Orange may specifically impact *BMAL1* expression in girls. In boys, however, there were no significant differences in dioxin exposure levels between the *BMAL1*-detected and -non-detected groups and no significant correlations between dioxin exposure and clock gene expression levels. These findings suggest that perinatal dioxin exposure may have a greater effect on clock gene expression in girls than in boys.

In children with *PER1* detection, perinatal TEQ-PCDF exposure was significantly associated with *PER1* expression; however, the directions of impact were different. *PER1* expression levels were higher in boys and lower in girls with higher perinatal TEQ-PCDF exposure, even after adjusting for maternal parity (a well-known confounder of dioxin levels in breast milk [11]). These findings suggest that sex differences in the pathways that regulate *PER1* transcription may be affected differently by PCDF exposure.

To the best of our knowledge, the present study is the first epidemiological investigation of the association between perinatal dioxin exposure and circadian clock gene expression in a dioxin-exposed population. However, several previous studies have reported the effects of TCDD exposure on clock gene expression in cells of the suprachiasmatic nucleus (SCN) in the brain or in the tissues of other organs in mice [3,4,17,18]. For example, Miller et al. (1999) reported that TCDD exposure reduces period circadian protein homolog 1 (PER1) and basic-helix-loop-helix ARNT-like protein 1 (BMAL1) protein levels in male mice [3]. Similarly, Garrett et al. (2006) reported that TCDD exposure decreases total *PER1* and *PER2* mRNA levels in murine hematopoietic precursors in mice [17]. However, TCDD exposure has also been reported to increase *PER1* expression in the mammary glands of mice [18], and Mukai et al. (2008) reported that TCDD exposure decreases *PER1* expression but increases *BMAL1* expression in the mouse SCN. In addition, Fader et al. (2019) reported that TCDD exposure induces either a decrease in amplitude or a complete loss of oscillation in the rhythmic expression of 15 hepatic core clock genes, including *BMAL1* and *PER1* [4], suggesting that TCDD-induced alterations in clock genes occur in tissues other than the SCN. Together, these results suggest that TCDD exposure may alter *BMAL1* and *PER1* (positive and negative regulators of circadian rhythm, respectively) not only in the SCN of the central nervous system but also in organs other than the brain, such as liver tissue, which is related to digestion and metabolism. In the future, we will follow-up with these children from the Da Nang birth cohort to investigate the impact of dioxin exposure on the expression of *PER1* and other clock genes that regulate *PER1* and control circadian rhythms in tissues from other organs.

### 4.2. Relationships Between Clock Gene Expression and Neurodevelopmental Symptoms, Sleep Durations, and ADHD-RS and C-SHARP Scores Examined at 8 Years of Age

In our previous study of children from the Da Nang birth cohort at 8 years of age, we reported that hyperactivity and covert aggression scores were significantly higher in children with high TCDD exposure, particularly in girls [6]. In the present study, we investigated sleep duration in these children and revealed that *BMAL1* expression was associated with TCDD and TEQ-PCDD/F exposure levels as well as decreased sleep duration at night and all sleep durations (including nap time) on weekdays, but increased sleep duration at night on holidays in girls. These findings suggest that increased *BMAL1* expression as a result of TCDD exposure may influence the sleep–wake rhythm to alter sleep durations; that is, children sleep late but wake up early because of going to school on weekdays, whereas they sleep late and wake up late on holidays. In girls, *BMAL1* expression levels were also correlated with ADHD-RS hyperactivity scores, which were significantly higher in the high TCDD and TEQ-PCDD/Fs groups in our previous study. Together, these results indicate that circadian clock genes, particularly *BMAL1*, may be a target of perinatal dioxin exposure that leads to decreased sleep duration and increased behavioral problems, which are often observed in children with ADHD.

In clinical studies, alterations in clock genes (particularly *BMAL1*) have been frequently identified in children with ADHD, who often have comorbid sleep disorders [9,19]. Baird et al. (2012) measured circadian rhythms in the oral mucosa of children and reported that *BMAL1* showed circadian rhythmicity in typically developing children. However, this rhythm is blunted in children with ADHD, which may lead to decreased duration and quality of sleep [20]. Duck et al. (2022) also reported a disturbed rhythmicity of *BMAL1* in children with ADHD, which was associated with alterations in sleep onset and duration [21]. These clinical results suggest that TCDD-induced alterations in the circadian system may be the core underlying pathology of the ADHD-like neurodevelopmental disorder observed in girls exposed to dioxins originating from Agent Orange [6].

In the present study, we demonstrated that higher *PER1* expression was associated with TEQ-PCDFs in boys in the *PER1*-detected group, which included boys without *BMAL1* detection. However, neither sleep durations nor any ADHD-RS and C-SHARP scores showed an association with *PER1* expression in this group.

In girls in the *PER1*-detected group, lower *PER1* expression levels were associated with higher TEQ-PCDF levels, whereas positive associations between *PER1* expression and verbal aggression and hostility scores were identified (i.e., there was lower aggression with lower *PER1* in girls with higher TEQ-PCDF exposure), which is inconsistent with the results of our previous study (higher aggression in girls with higher TEQ-PCDD/Fs exposure) [6]. Moreover, no association was found between *PER1* expression and sleep duration or ADHD-RS scores were noted among them. Together, our findings indicate that lower *PER1* expression with higher TEQ-PCDFs might activate other factors related to aggression because *PER1* plays a key role in the secretion of corticosterone and testosterone, whose levels are related to aggression [9].

### 4.3. Possible Mechanisms of Dioxin Exposure-Induced Alterations of Circadian Rhythms

The biological effects of dioxins, particularly TCDD, are primarily exerted through their binding to the aryl hydrocarbon receptor (AhR). The AhR belongs to the basic-helix-loop-helix (bHLH)/Per-Arnt-Sim (PAS) family of heterodimeric transcription factors, and can form a complex with aryl hydrocarbon receptor nuclear translocator (ARNT) to mediate the expression of a diverse set of genes through dioxin-responsive elements in the promoter regions of target genes [22]. The AhR, ARNT, circadian locomoter output cycles protein kaput (CLOCK), and BMAL1 proteins are members of the bHLH/PAS domain–containing family of key proteins, which play a critical role in both development and adaptation to the environment [23]. Moreover, AhR and ARNT are highly homologous to the core circadian clock gene proteins, including CLOCK and BMAL1. It has been hypothesized that AhR and ARNT may functionally interact with members of the circadian clock gene family. Notably, disruption of circadian clock genes, which may alter the dioxin-mediated AhR signaling pathway [18], has been reported in a dioxin-exposed animal study [24]. These findings indicate that the possible interplay between dioxin-mediated AhR signaling and clock gene expression [25] might lead to alterations in typical light/dark cycles or behaviors.

Mukai et al. (2008) reported that exposure to TCDD alters the expression of core circadian genes (*Per1* and *Bmal1*) in the mouse SCN [5]. Furthermore, peripheral oscillations in clock genes, such as those occurring in buccal cells, reportedly originate from the SCN via mainly neuronal and hormonal cues [26]. TCDD exposure has been reported to affect circadian thyroid hormone rhythms [27], reduce axonal growth [28], disrupt dendritic growth and neuronal connections in the white matter of various brain regions (particularly the hippocampus and amygdala) [29], and alter synaptic transmission and plasticity [30,31]. Taken together, these findings indicate that the effects of exposure to dioxins, particularly TCDD, may impact not only central clock genes in the SCN but also the communication between peripheral oscillations and the central clock. In addition, there are reportedly impairments in reproductive hormones—leading to irregular menstrual cycles, endometriosis, and infertility—in women undertaking shift work, which disrupts the circadian program [32]. This suggests that circadian clock genes, particularly *BMAL1*, may be important targets underlying the endocrine rupture effects of TCDD.

### 4.4. Sex Different Effects of Perinatal Dioxin Exposure on Neurodevelopment

In the current study, the effects of perinatal TCDD and TEQ-PCDD/F exposure on circadian clock gene expression associated with sleep and behavior problems were found only in girls, suggesting female-specific effects on circadian rhythm. In our previous studies investigating associations between dioxin exposure markers and neurodevelopment in children aged 4 months to 5 years of age from the Da Nang cohort, we reported significant impacts of perinatal dioxin exposure on neurodevelopment, particularly in boys, although increased autistic traits were also found in girls with high TCDD [12]. However, at 8 years of age, increased ADHD traits associated with increased TEQ-PCDD/Fs and TCDD exposure were found only in girls, whereas poor learning ability associated with dioxin exposure was observed in boys [13]. We also reported that perinatal TCDD exposure altered mirror neuron activity in the brain at 9 years of age, which plays an important role in social–emotional behavior, only in girls [33]. These results suggest that the effects of perinatal dioxin exposure on neurodevelopment were marked in boys during infancy and early childhood before school age, whereas the effects in girls were limited to specific domains of neurodevelopment, such as social–emotional behavior, and became remarkable after reaching school age.

MRI imaging studies for infants who were born prematurely and at higher risk for neurodevelopmental deficits reported sex differences in brain structure, higher cortical gray matter volume in girls, higher white matter volume in boys, and significant effects of gestational age on cortical volume only in boys [34]. These findings suggest that sex differences in the development of the brain during infancy and the gestational period have more robust impacts on the brains of males born prematurely, leading to poor neurodevelopment in later life. Kurth et al. (2021) [35] performed MRI analysis in children aged 6–17 years to estimate brain sex based on the difference in brain structure using multivariate machine learning and successfully discriminated male brains from female brains, even in children aged 6 years. They also found that the effect size of sex increased with age, suggesting the largest difference in the brain between boys and girls aged 17 years. These findings indicate that sex differences in brain structure exist during childhood before puberty and subsequently increase during adolescence.

Taken together, the developing brain of boys is suspected to be more vulnerable than that of girls from the perinatal period to adolescence (lower risks in girls, in other words), which might be related to sex differences in the effects of perinatal dioxin exposure on neurodevelopment in Vietnamese children. In girls, the affected domain of neurodevelopment was limited to social−emotional behavior, which was impaired in children with ADHD, and the effects became more remarkable with age compared with boys. Further studies on the effects of dioxin on brain structure and functions associated with altered behavior in adolescence are necessary to clarify the sex differences in the effects on the developing brain.

### 4.5. Strengths and Limitations

The present study revealed the impact of perinatal dioxin exposure on circadian rhythms, as indicated by the expression of clock genes in children. It also examined the neurodevelopment of these children, including ADHD symptoms. The associations between sleep duration and clock gene alterations caused by dioxin exposure were also investigated, and significant effects on sleep–wake rhythms were observed. However, one major limitation of the present study is its small sample size in terms of *BMAL1* detection; only simple correlations between *BMAl1* expression levels and perinatal dioxin exposure or neurodevelopmental symptoms could be performed. This may be because buccal samples were collected during the day, when the transcriptional activity of *BMAL1* was relatively low. In the future, sampling from this cohort of children should occur at night, when higher *BMAL1* expression is expected; in this way, clock genes, including *BMAL1* and *CLOCK,* can be accurately measured to confirm the effects of dioxin on circadian rhythms. Another limitation of the present study is the lack of data on polychlorinated biphenyl levels, which have been previously reported to affect circadian rhythms [32]. In the future, we will attempt to investigate the impact of perinatal dioxin exposure on circadian clock gene expression in children from the Bien Hoa cohort, whose dioxin-like polychlorinated biphenyl exposure levels were measured in maternal milk samples.

## 5. Conclusions

Higher perinatal TCDD and TEQ-PCDD/F exposure levels were associated with lower *BMAL1* expression, which was further associated with shorter sleep duration and higher ADHD-RS hyperactivity scores in girls. In contrast, higher perinatal TEQ-PCDF exposure was associated with higher *PER1* expression in girls but lower expression in boys. However, lower *PER1* expression levels were not associated with sleep duration or ADHD-RS scores in either sex. A follow-up of children from our birth cohorts in Vietnam is needed to evaluate the associations between dioxin exposure and the expression of clock genes, including *BMAL1* and *PER1,* in buccal cells collected at night and during the day.

## Figures and Tables

**Table 1:** Subject characteristics and sleep duration.

		Boys (N = 56)		Girls (N = 34)		
	Units	Mean, [N]	SD, [%]	Mean, [N]	SD, [%]	
Birthweight	g	3304.5	439.8	3113.2	386.0	
Gestational weeks	weeks	39.6	0.8	39.5	0.8	
Maternal age at birth	years	28.9	5.9	28.1	6.7	
Parity of mothers	% primiparae	[11]	[19.6]	[12]	[34.3]	
Maternal education	years	8.7	3.5	8.1	3.4	
Age at the survey	months	105.0	1.7	105.7	1.7	
Weight at the survey	kg	31.8	8.7	30.2	8.1	
Height at the survey	cm	130.7	6.2	130.5	5.4	
BMI at the survey	kg/m^2^	18.4	3.7	17.5	3.8	
*Weekdays*						
Sleep duration at night	hours	8.5	0.6	8.5	0.8	
Nap duration	minutes	106.8	37.8	81.2	46.6	*
Total sleep duration	hours	10.1	0.9	9.8	1.1	
*Holydays*						
Sleep duration at night	hours	9.0	0.9	9.5	1.1	*
Nap duration	minutes	84.8	64.3	78.5	59.1	
Total sleep duration	hours	10.3	1.4	10.7	1.6	

* *p* < 0.05 between boys and girls (independent sample *t*-test); BMI: body mass index; N: number of subjects; SD: standard deviation.

**Table 2:** Comparison of perinatal dioxin levels between children with and without *BMAL1* detection.

BMAL1	Non-Detected		Detected		
	Mean	SD	Mean	SD	
Boys	N = 40		N = 16		
TCDD	1.24	2.05	1.32	2.15	
TEQ−PCDDs	7.20	1.60	7.13	1.56	
TEQ−PCDFs	5.62	1.52	4.87	1.60	
TEQ−PCDD/Fs	12.9	1.54	12.1	1.55	
Girls	N = 19		N = 15		
TCDD	1.30	2.66	2.38	1.79	*
TEQ−PCDDs	6.82	1.86	9.71	1.39	
TEQ−PCDFs	5.04	1.78	8.05	1.38	**
TEQ−PCDD/Fs	12.0	1.78	17.9	1.36	*

* *p* < 0.05, ** *p* < 0.01; N: number of subjects; PCDD: polychlorinated dibenzodioxin; PCDF: polychlorinated dibenzofuran; TCDD: 2,3,7,8-tetrachlorodibenzo-p-dioxin; TEQ: toxic equivalent.

**Table 3:** Correlations (Spearman’s rho) between relative expression levels of clock genes and perinatal dioxin exposure in children with *BMAL1* detection.

	BMAL1		PER1		CRY1	CRY2
Boys (N = 16)						
TCDD	−0.209		0.062		−0.096	−0.329
TEQ−PCDDs	−0.174		0.129		−0.061	−0.329
TEQ−PCDFs	−0.059		0.003		−0.114	−0.391
TEQ−PCDD/Fs	−0.185		0.059		−0.121	−0.403
Girls (N = 15)						
TCDD	0.529	*	−0.168		0.096	-0.016
TEQ−PCDDs	0.418		−0.221		0.168	0.082
TEQ−PCDFs	0.393		−0.564	*	0.168	−0.115
TEQ−PCDD/Fs	0.525	*	−0.339		0.236	−0.016

* *p* < 0.05; N: number of subjects (note, boys: N = 15 for *CRY1* and girls: N = 13 for *CRY2*); PCDD: polychlorinated dibenzodioxin; PCDF: polychlorinated dibenzofuran; TCDD: 2,3,7,8-tetrachlorodibenzo-p-dioxin; TEQ: toxic equivalent.

**Table 4:** Correlations (Spearman’s rho) between relative expression levels of clock genes and sleep duration in children with *BMAL1* detection.

	*Weekdays*						*Holydays*			
	Night Sleep		Nap		All Sleep		Night Sleep		Nap	All Sleep
Boys (N = 16)										
BMAL1	0.071		−0.273		−0.308		−0.142		0.111	−0.041
PER1	−0.086		−0.271		0.083		0.267		0.230	0.311
CRY1	−0.322		−0.080		−0.020		0.071		−0.260	−0.222
CRY2	−0.204		−0.356		−0.219		0.241		0.053	0.119
Girls (N = 15)										
BMAL1	−0.532	*	−0.483		−0.640	*	0.585	*	−0.006	0.368
PER1	−0.183		0.345		0.161		−0.402		−0.143	−0.336
CRY1	−0.601	*	0.330		−0.158		0.039		0.378	0.403
CRY2	−0.218		0.696	**	0.354		−0.285		0.264	0.089

* *p* < 0.05, ** *p* < 0.01; N: number of subjects (note, boys: N = 15 for *CRY1* and girls: N = 13 for *CRY2*).

**Table 5:** Correlations (Spearman’s rho) between relative expression levels of clock genes and ADHD-RS and C-SHARP scores in children with *BMAL1* detection.

	ADHD-RS						C-SHARP						
	Inattention		Hyperactivity		ADHD		Verbal	Bulling	Covert	Hostility		Physical	
Boys (N = 16)													
BMAL1	0.247		0.366		0.270		0.182	0.437	0.010	0.457		0.519	*
PER1	0.534	*	0.373		0.392		0.364	0.127	−0.004	0.474		0.385	
CRY1	0.413		0.146		0.199		0.470	0.353	0.394	0.491		0.561	*
CRY2	0.563	*	0.465		0.503	*	0.355	0.314	−0.016	0.756	**	0.460	
Girls (N = 15)													
BMAL1	0.234		0.520	*	0.334		0.248	0.247	0.128	0.044		0.337	
PER1	0.343		−0.624	*	−0.489		−0.175	−0.383	−0.265	−0.039		−0.452	
CRY1	−0.322		0.054		−0.138		0.118	−0.136	0.141	0.000		−0.115	
CRY2	−0.577	*	−0.247		−0.411		−0.312	−0.384	−0.222	−0.228		−0.390	

* *p* < 0.05, ** *p* < 0.01; ADHD: attention deficit hyperactivity disorder; C-SHARP: Children’s Scale of Hostility and Aggression: Reactive/Proactive; N: number of subjects (note, boys: N = 15 for *CRY1* and girls: N = 13 for *CRY2*); RS: rating scale.

**Table 6:** Maternal parity–adjusted associations between relative expression levels of clock genes and perinatal dioxin exposure in children with *PER1* detection.

	PER1				CRY1		CRY2	
	ρ		β		ρ	β	ρ	β
Boys	(N = 56)				(N = 32)		(N = 41)	
TCDD	0.094		0.051		−0.069	0.054	−0.041	0.027
TEQ−PCDDs	0.194		0.202		−0.054	−0.028	0.021	0.100
TEQ−PCDFs	0.280	*	0.300	*	0.021	0.005	0.135	0.174
TEQ−PCDD/Fs	0.241		0.255		−0.033	−0.017	0.069	0.137
Girls	(N = 34)				(N = 21)		(N = 31)	
TCDD	−0.126		−0.074		−0.174	−0.196	−0.057	−0.111
TEQ−PCDDs	−0.276		−0.152		−0.127	−0.030	−0.085	−0.065
TEQ−PCDFs	−0.499	**	−0.394	*	−0.153	−0.038	−0.266	−0.206
TEQ−PCDD/Fs	−0.385	*	−0.259		−0.142	−0.033	−0.179	−0.127

* *p* < 0.05; ** *p* < 0.01; ρ: Sperman’s correlation; β: standardized coefficients after adjusting for maternal parity categories (primipara/multipara); PCDD: polychlorinated dibenzodioxin; PCDF: polychlorinated dibenzofuran; TCDD: 2,3,7,8-tetrachlorodibenzo-p-dioxin; TEQ: toxic equivalent.

**Table 7:** Maternal education–adjusted associations between relative expression levels of clock genes and ADHD-RS and C-SHARP scores in children with *PER1* detection.

		ADHD-RS			C-SHARP						
		Inattention	Hyperactivity	ADHD	Verbal		Bulling	Covert	Hostility		Physical
Boys	ρ	0.022	−0.101	−0.088	0.044		−0.136	−0.044	0.002		0.073
(N = 56)	β	0.081	−0.140	−0.029	−0.034		−0.128	−0.148	0.042		0.075
Girls	ρ	−0.051	−0.301	−0.165	0.507	**	0.111	0.299	0.355	*	0.153
(N = 34)	β	−0.066	−0.258	−0.164	0.575	**	0.303	0.336	0.423	*	0.160

* *p* < 0.05; ** *p* < 0.01; ρ: Sperman’s correlation; β: standardized coefficients after adjusting for maternal education (years); ADHD: attention deficit hyperactivity disorder; C-SHARP: Children’s Scale of Hostility and Aggression: Reactive/Proactive; N: number of subjects; RS: rating scale.

## Data Availability

The data presented in this study are available upon request from the corresponding author. The data are not publicly available due to privacy or ethical restrictions.
